# eGFR slope improvement with SGLT2 inhibitors is not statistically associated with changes in urinary protein in Japanese CKD patients: a single-center real-world analysis

**DOI:** 10.1007/s10157-026-02860-7

**Published:** 2026-04-13

**Authors:** Shogo Kuwagata, Masami Chin-Kanasaki, Aki Yamada, Tomonori Sakae, Nahomi Ishimoto, Yoshimi Imamura-Uehara, Sho Sugahara, Kosuke Yamahara, Mako Yasuda-Yamahara, Yuki Tanaka-Sasaki, Itsuko Miyazawa, Shinji Kume

**Affiliations:** 1 Department of Diabetes, Endocrinology and Nephrology, Shiga University of Medical Science, Tsukinowa-cho, Seta, Otsu, Shiga 520-2192 Japan; 2 Center for Medical Data Science (CMeDS), Shiga University of Medical Science, Tsukinowa-Cho, Seta, Otsu, Shiga 520-2192 Japan

**Keywords:** Sodium–glucose cotransporter 2 inhibitor, Dapagliflozin, Chronic kidney disease, EGFR slope, Urinary protein, Real-world study

## Abstract

**Background:**

SGLT2 inhibitors reduce urinary protein and slow eGFR decline in clinical trials. However, real-world evidence on within-patient changes in eGFR slope before and after treatment initiation, and their relationship to changes in urinary protein, remains limited. This study was designed to address these concerns.

**Methods:**

This retrospective study included 90 Japanese CKD patients who initiated dapagliflozin and had ≥ 5 eGFR measurements during both the 12-month periods before and after initiation. The treatment effect was defined as the ΔeGFR slope (post-minus pre-initiation slope). Clinical factors associated with ΔeGFR slope were assessed using multivariable regression.

**Results:**

The mean eGFR slope significantly improved from − 5.12 ± 5.93 to 1.59 ± 5.27 mL/min/1.73 m^2^/year (*p* < 0.0001). Urinary protein decreased modestly without statistical significance (median 0.65 − 0.55 g/gCr, *p* = 0.155). Significant improvements in eGFR slope were observed regardless of baseline urinary protein level or the reduction rate. Multivariable analysis revealed that a reduction in serum uric acid (*β* =  − 0.26, *p* < 0.001), but not urinary protein, was independently associated with the ΔeGFR slope.

**Conclusions:**

Dapagliflozin significantly improves the eGFR slope, which was not statistically associated with urinary protein changes in this cohort, in routine clinical practice. These findings underscore the clinical value of eGFR slope-based evaluation for monitoring individualized therapeutic responses.

## Introduction

Sodium–glucose cotransporter 2 (SGLT2) inhibitors have demonstrated robust kidney and cardiovascular benefits in patients with chronic kidney disease (CKD) across a broad range of estimated glomerular filtration rate (eGFR) and albuminuria levels in large randomized-controlled trials (RCTs), including CREDENCE, DAPA-CKD, and EMPA-KIDNEY [1–3]. Based on this evidence, the Kidney Disease: Improving Global Outcomes (KDIGO) 2024 guidelines recommend SGLT2 inhibitors as a foundational therapy for CKD patients at high risk of progression, irrespective of diabetes status or baseline albuminuria [4].

Traditional CKD clinical trials have relied on "hard" endpoints, such as a doubling of serum creatinine, progression to kidney replacement therapy, or a sustained 40–50% decline in eGFR [5]. However, because these events often occur late in the disease course, they require large sample sizes and prolonged follow-up. To address these limitations, the eGFR slope has been validated as a reliable surrogate endpoint reflecting the trajectory of kidney function decline [6, 7]. Meta-analyses have shown that the chronic eGFR slope, which excludes early hemodynamic effects (the "initial dip"), accurately predicts long-term kidney outcomes [6, 7]. More recently, the within-patient change in eGFR slope before and after treatment (the ΔeGFR slope) has been proposed as a sensitive approach to detect therapeutic effects in short-term clinical settings [8]. While RCTs of SGLT2 inhibitors have consistently shown attenuated chronic eGFR slopes in treatment groups compared with placebo, these trials do not capture pre-treatment slope values within individuals [9]. Consequently, it remains uncertain whether SGLT2 inhibition actually improves the eGFR slope at the individual-patient level in real-world practice.

Changes in albuminuria have also served as a surrogate marker for CKD progression [10]. Although SGLT2 inhibitors significantly reduce urinary protein, post hoc analyses suggest that they slow eGFR decline even in patients with low baseline albuminuria, implying the presence of urinary protein-independent protective mechanisms [3]. Because RCTs lack individual pre-treatment eGFR slope data, the specific link between an individual’s treatment-induced change in urinary protein and their subsequent improvement in eGFR slope remains poorly defined.

Therefore, the primary objective of this study was to determine whether SGLT2 inhibition improves the eGFR slope at the individual-patient level in routine care and to evaluate the extent to which changes in urinary protein account for this improvement. To address this, we conducted a single-center retrospective study of Japanese patients with CKD, analyzing within-patient changes in eGFR slope before and after initiation of dapagliflozin (ΔeGFR slope) and their association with changes in urinary protein. Our findings suggest that SGLT2 inhibitors improve the eGFR slope, which was not statistically associated with changes in urinary protein in this cohort, highlighting the value of slope-based assessment for individualized therapeutic evaluation.

## Materials and methods

### Study design and population

**Fig. 1:**
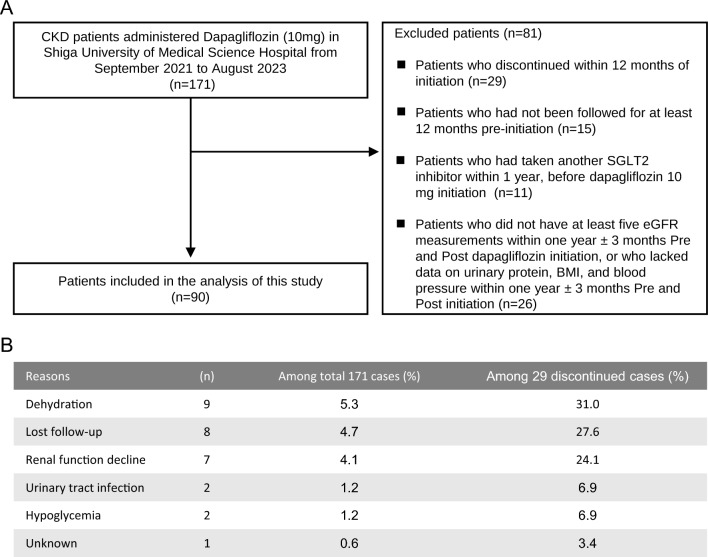
Flow diagram of patient selection and reasons for SGLT2 inhibitor discontinuation. **A** Patient selection flow diagram **B** Reasons for discontinuation of SGLT2 inhibitors and their proportions in the overall cohort (*n* = 171) and among discontinued cases (*n* = 29)

This retrospective cohort study was conducted at Shiga University of Medical Science Hospital. We identified patients with CKD who were prescribed the SGLT2 inhibitor dapagliflozin (10 mg/day) between September 2021 and August 2023. Of the 171 patients initially screened Fig. 1A, 81 were excluded based on the following criteria:

- ・Discontinuation of the SGLT2 inhibitor within 12 months of initiation (*n* = 29). Because this study was a retrospective observational study based on medical records, and no standardized discontinuation criteria were defined, discontinuation was determined by attending physicians in routine clinical practice, with reasons detailed in Fig. 1B**.**
- ・Follow-up period of less than 12 months prior to SGLT2 inhibitor initiation (*n* = 15).
- ・Use of other SGLT2 inhibitors within 12 months before dapagliflozin initiation (*n* = 11).
- ・Fewer than five eGFR measurements during either the 12-month pre- or post-initiation periods, or missing clinical data (urinary protein, BMI, or blood pressure) within ±3 months of the study periods (*n* = 26).
- A total of 90 patients were included in the final analysis. The study protocol was approved by the Ethics Committee of Shiga University of Medical Science (Approval No. R2024-073). The requirement for individual informed consent was waived due to the study’s retrospective nature and the use of anonymized data.

### Data collection

Baseline characteristics and laboratory data were extracted from electronic medical records. eGFR was calculated using the Japanese coefficient-modified equation for estimated glomerular filtration rate [11].

eGFR (mL/min/1.73 m^2^) = 194 × (Serum creatinine) ^− 1.094^ × (Age) ^− 0.287^ × 0.739 (if female).

This equation was established to improve accuracy in the Japanese population. All available eGFR measurements were collected during the 12 months before and after initiation of an SGLT2 inhibitor. CKD stages and risk categories were classified using the KDIGO "heat map" based on baseline eGFR and urinary protein. Urinary protein was expressed as g/gCr and categorized into < 0.15 (A1), 0.15–0.49 (A2), and ≥ 0.5 (A3).

### Estimation of eGFR slope

eGFR trajectories were analyzed using piecewise linear mixed-effects models with separate slopes for − 12 to 0 months (pre) and 2–12 months (post). Fixed effects included pre-time, post-time, and a post-period indicator allowing a discontinuity at month 2. Random effects consisted of subject-specific intercepts and time slopes. An unstructured covariance matrix was assumed, and parameters were estimated using restricted maximum likelihood (REML). Analyses were performed in Python (statsmodels MixedLM). Two-sided 95% confidence intervals were calculated for all fixed-effect estimates.

### Other outcome measures

Body mass index (BMI), hemoglobin A1c (HbA1c), systolic blood pressure (SBP), low-density lipoprotein (LDL) cholesterol, serum uric acid, hematocrit, and urinary red blood cell (U-RBC) counts were evaluated at baseline and as changes from the pre- to post-treatment periods. Urinary protein was assessed at baseline and summarized as the percentage change from the pre- to post-treatment period. Concomitant medications, including renin–angiotensin system inhibitors (RASis), mineralocorticoid receptor antagonists (MRAs), glucagon-like peptide-1 receptor agonists (GLP-1RAs), urate-lowering drugs (ULDs), and erythropoiesis-stimulating agents (ESAs), were assessed based on their use before SGLT2 inhibitor initiation.

### Statistical analysis

Continuous variables were compared using Student’s t test or the Wilcoxon rank-sum test for non-normally distributed data, such as urinary protein levels and urinary red blood cell counts. Categorical variables were analyzed using the Chi-square test. To identify clinical factors associated with the ΔeGFR slope, univariable and multivariable linear regression analyses were performed with the ΔeGFR slope as the dependent variable. We reported unstandardized β coefficients for univariable analyses and standardized *β* coefficients for multivariable analyses to allow for comparison of effect sizes among covariates. Furthermore, Pearson’s correlation was used to assess linear association between the ΔeGFR slope and three key variables: the percentage change in urinary protein, baseline serum uric acid levels, and the change in serum uric acid levels. For all analyses, 95% confidence intervals (CIs) were calculated, and a two-sided p value < 0.05 was considered statistically significant.

## Results

### Baseline characteristics

The final study cohort comprised 90 patients with chronic kidney disease (CKD) who had newly initiated dapagliflozin, an SGLT2 inhibitor, and met all eligibility criteria. The distribution of CKD stages at the treatment initiation is summarized in Table 1. The majority of patients were classified as having CKD stages 3 or 4. Specifically, a substantial proportion of participants had an eGFR of 30–59 mL/min/1.73 m^2^ and presented with heavy urinary protein (≥ 0.5 g/gCr). Detailed baseline patient characteristics are presented in Table 2. The mean age of the cohort was 60.9 ± 15.1 years, and 52 patients (57.8%) were male. The mean baseline eGFR was 44.2 ± 18.6 mL/min/1.73 m^2^. Regarding comorbidities and concomitant therapies, 21 patients (23.3%) had diabetes mellitus, and 65 patients (72.2%) were receiving RASis at baseline. The cause of CKD was most commonly chronic glomerulonephritis (42 cases, 46.7%), followed by diabetes-related kidney disease (21 cases, 23.3%) and nephrosclerosis (15 cases, 16.7%).

**Table 1:** The distribution of patients by CKD classification before SGLT2 inhibitor therapy (*n* = 90)

			A1	A2	A3
Proteinuria (g/gCr)			< 0.15	0.15–0.49	≥ 0.5
GFR (mL/min/1.73 m^2^)	G1	≥ 90	0 (0%)	1 (1.1%)	3 (3.3%)
	G2	60–89	0 (0%)	2 (2.2%)	11 (12.2%)
	G3a	45–59	4 (4.4%)	5 (5.6%)	10 (11.1%)
	G3b	30–44	6 (6.7%)	15 (16.7%)	14 (15.6%)
	G4	15–29	1 (1.1%)	3 (3.3%)	15 (16.7%)
	G5	< 15	0 (0%)	0 (0%)	0 (0%)

Number (percentage) of patients in each CKD severity category. Albuminuria categories were approximated using urinary protein/creatinine ratio thresholds (g/gCr). *SGLT2* inhibitor sodium–glucose cotransporter 2 inhibitor, *eGFR *estimated glomerular filtration rate

**Table 2:** Clinical characteristics before and after SGLT2 inhibitor therapy

	Before (*n* = 90)	Analyzed sample size (*n*)	After (*n* = 90)	Analyzed sample size (*n*)	*p* value
Patient characteristics					
Age (years)	60.9 ± 15.1	*n* = 90			
Sex (male:female)	52: 38	*n* = 90			
Prevalence of DM (%)	23.3	*n* = 90			
BMI (kg/m^2^)	24.4 ± 4.57	*n* = 90	22.8 ± 4.60	*n* = 90	< 0.0001
SBP (mmHg)	131.6 ± 15.5	*n* = 90	127.8 ± 14.3	*n* = 90	0.015
Laboratory data					
HbA1c (%)	6.92 ± 1.34	*n* = 33	6.88 ± 1.21	*n* = 33	0.84
LDL-C (mg/dL)	104.4 ± 26.8	*n* = 74	100.6 ± 26.17	*n* = 74	0.18
Uric acid (mg/dL)	6.16 ± 1.29	*n* = 90	5.38 ± 1.23	*n* = 90	< 0.0001
Hematocrit (%)	39.8 ± 4.64	*n* = 90	42.3 ± 5.82	*n* = 90	< 0.0001
eGFR (mL/min/1.73m^2^)	44.2 ± 18.6	*n* = 90	42.8 ± 18.2	*n* = 90	0.054
eGFR slope (mL/min/1.73m^2^/year)	−5.12 ± 5.93	*n* = 90	1.59 ± 5.27	*n* = 90	< 0.0001
Urinary protein (g/gCr)	0.65 (0.28—1.48)	*n* = 90	0.55 (0.23—1.32)	*n* = 90	0.155
U-RBC > 20/HPF (%)	10.0	*n* = 90	4.4	*n* = 90	0.25
Medications					
RAS inhibitors (%)	72.2	*n* = 90	69.8	*n* = 90	0.62
MRAs (%)	1.1	*n* = 90	1.10	*n* = 90	1.0
GLP-1 RAs (%)	2.20	*n* = 90	5.60	*n* = 90	0.25
ULDs (%)	37.8	*n* = 90	41.1	*n* = 90	0.65
ESAs (%)	0.00	*n* = 90	0.00	*n* = 90	1.0
Causes of CKD					
Diabetic kidney disease (%)	23.3	*n* = 90			
Chronic glomerular nephritis (%)	46.7	*n* = 90			
Nephrosclerosis (%)	16.7	*n* = 90			
Tubulointerstitial diseases (%)	1.1	*n* = 90			
Others & unknown (%)	12.2	*n* = 90			

Values are presented as mean ± SD or median (IQR)

*SGLT2* inhibitor sodium–glucose cotransporter 2 inhibitor, *DM* diabetes mellitus, *BMI* body mass index, *HbA1c* hemoglobin A1c, *SBP* systolic blood pressure, *LDL-C* low-density lipoprotein cholesterol, *eGFR* estimated glomerular filtration rate, *U-RBC* urinary red blood cells per high-power field, *RAS* inhibitors renin–angiotensin system inhibitors, *MRAs* mineralocorticoid receptor antagonists, *GLP*-*1RAs* glucagon-like peptide-1 receptor agonists, *ULDs* uric acid-lowering drugs, *ESAs* erythropoiesis-stimulating agents

### Changes in clinical characteristics after dapagliflozin initiation

Changes in clinical and laboratory parameters before and after dapagliflozin initiation are summarized in Table 2. Body mass index decreased significantly from 24.4 ± 4.57 to 22.8 ± 4.60 kg/m^2^ (*p* < 0.0001). Similarly, systolic blood pressure was reduced from 131.6 ± 15.5 to 127.8 ± 14.3 mmHg (*p* = 0.015). Notably, serum uric acid levels decreased significantly from 6.16 ± 1.29 − 5.38 ± 1.23 mg/dL (*p* < 0.0001), while hematocrit increased significantly from 39.8 ± 4.64% to 42.3 ± 5.82% (*p* < 0.0001). Hemoglobin A1c remained stable in the subset of patients with available data (6.92 ± 1.34% to 6.88 ± 1.21%, *p* = 0.84, *n* = 33). There were no significant differences in the prescription rates of RASis, GLP-1RAs, ULDs, or ESAs during the observation period (Table 2**)**.

### Changes in renal parameters after dapagliflozin initiation

**Fig. 2:**
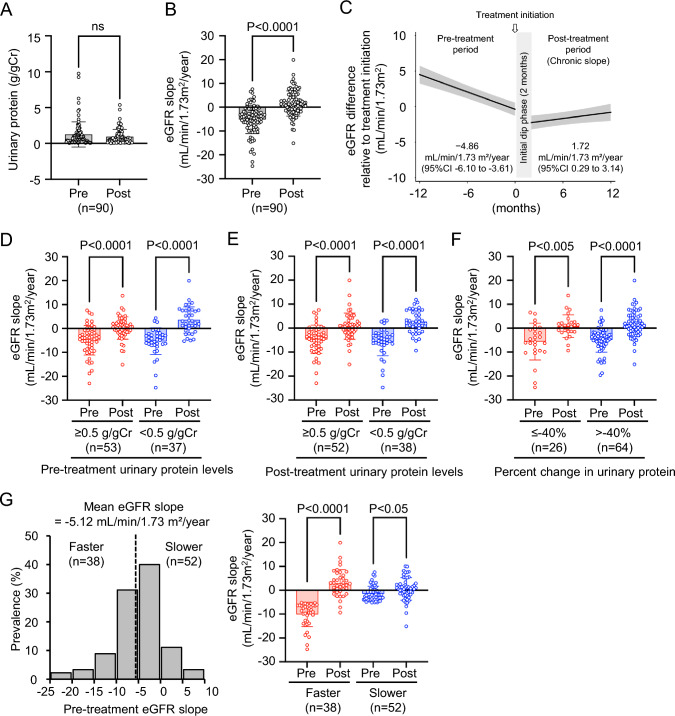
Relationship between changes in eGFR slope and urinary protein during SGLT2 inhibitor treatment. **A** Comparison of urinary protein levels in the pre-treatment and post-treatment periods. **B** Comparison of eGFR slopes in the pre-treatment and post-treatment periods. **C** Piecewise linear mixed-effects model analysis of eGFR over the 12-month pre-treatment and post-treatment periods. The solid line represents the estimated mean eGFR trajectory, and the gray shaded area indicates the 95% confidence interval. **D** Comparison of pre- and post-treatment eGFR slopes stratified by baseline urinary protein levels (≥ 0.5 vs < 0.5 g/gCr). **E** Comparison of pre- and post-treatment eGFR slopes stratified by urinary protein levels at 1 year after treatment initiation (≥ 0.5 vs < 0.5 g/gCr). **F** Comparison of pre- and post-treatment eGFR slopes according to change in urinary protein (≤ − 40% vs >  − 40%). **G** Comparison of pre- and post-treatment eGFR slopes in patients stratified by the mean pre-treatment eGFR slope (− 5.12 mL/min/1.73 m^2^/year), showing similar improvements in both the faster-decline and slower-decline groups

The median urinary protein level decreased from 0.65 (interquartile range [IQR], 0.28–1.48) to 0.55 (IQR, 0.23–1.32) g/gCr; however, this change did not reach statistical significance (*p* = 0.155; Fig. 2A. In contrast, the eGFR trajectory showed substantial improvement. The mean pre-treatment eGFR slope was − 5.12 ± 5.93 mL/min/1.73 m^2^/year. In the linear mixed-effects model, the estimated pre-treatment slope was − 4.86 mL/min/1.73 m^2^/year (95% CI − 6.10 to − 3.61) Fig. 2B and C. Following dapagliflozin initiation, the mean post-treatment (chronic phase) eGFR slope significantly improved to 1.59 ± 5.27 mL/min/1.73 m^2^/year. The corresponding model-estimated slope was 1.72 mL/min/1.73 m^2^/year (95% CI, 0.29 to 3.14; *p* < 0.0001) Fig. 2B and C. This represents a significant attenuation of renal function decline, resulting in a mean ΔeGFR slope of 6.71 mL/min/1.73 m^2^/year. The median number of eGFR measurements per patient was 6 (IQR, 5–8) during the pre-treatment period and 6 (IQR, 5–7.25) during the post-treatment period. To examine whether the observed improvement could be explained solely by regression to the mean, patients were stratified according to whether their pre-treatment eGFR slope was below (faster decline, *n* = 38) or above (slower decline, *n* = 52) the cohort mean. Significant improvements in post-treatment slopes were observed in both groups Fig. 2G, indicating that the observed treatment effect was not restricted to individuals with steep baseline decline.

### Urinary protein-stratified analysis of ΔeGFR slope

To further investigate whether changes in urinary protein mediated the observed benefits in eGFR slope, patients were stratified by baseline and post-treatment urinary protein categories (≥ 0.5 g/gCr vs. < 0.5 g/gCr). Dapagliflozin significantly improved eGFR slopes in both baseline categories Fig. 2D and both post-treatment categories Fig. 2E. Specifically, the improvement was evident even in patients who maintained high levels of urinary protein (≥ 0.5 g/gCr) after 1 year of treatment (*p* < 0.0001; Fig. 2E. Similarly, when stratified by the magnitude of urinary protein reduction, significant eGFR slope improvements were observed not only in the group with ≥ 40% reduction but also in the subgroup with < 40% reduction (*p* < 0.0001; Fig. 2F. Collectively, these findings indicate that the renoprotective effect of dapagliflozin, as reflected by the ΔeGFR slope, was not statistically associated with baseline urinary protein severity or with treatment-related changes in urinary protein in this cohort.

### Clinical factors associated with ΔeGFR slope

Univariable and multivariable linear regression analyses were performed to identify clinical variables associated with the ΔeGFR slope. In univariable analyses, significant associations with ΔeGFR slope were observed for baseline serum uric acid, baseline hematocrit, pre-treatment eGFR slope, change in BMI, and change in serum uric acid. Specifically, higher baseline serum uric acid levels (*β* = 1.64; 95% CI 0.28–3.00; *p* = 0.02), lower baseline hematocrit (*β* =  − 0.41; 95% CI − 0.79 to 0.03; *p* = 0.04), steeper pre-treatment eGFR slope (*β* =  − 1.14; 95% CI − 1.33 to − 0.96; *p* < 0.001), greater reduction in BMI (*β* =  − 2.18; 95% CI − 3.64 to − 0.71; *p* = 0.004), and greater reduction in serum uric acid (*β* =  − 4.42; 95% CI − 5.89 to − 2.95; *p* < 0.001) were significantly associated with improvement in ΔeGFR slope (Table 3).

**Table 3:** Univariable and multivariable regression analyses of the association between ΔeGFR slope before and after SGLT2 inhibitor treatment and clinical variables

Variables	Univariable *β* (95%CI)	*p* value	Multivariable Standardized *β* (95%CI)	*p* value
Age (years)	0.07 (− 0.05 to 0.18)	0.28	0.03 (− 0.07 to 0.10)	0.66
Sex (male:female)	0.06 (− 3.59 to 3.70)	0.98	0.03 (− 2.36 to 3.34)	0.73
BMI (kg/m^2^)	0.13 (− 0.26 to 0.53)	0.50		
Prevalence of DM (%)	− 0.65 (− 3.61 to 4.90)	0.76		
HbA1c (%)	− 0.51 (− 3.31 to 2.28)	0.71		
SBP (mmHg)	0.02 (− 0.10 to 0.14)	0.75		
LDL cholesterol** (**mg/dL)	− 0.03 (− 0.10 to 0.05)	0.48		
Uric acid (mg/dL)	1.64 (0.28 to 3.00)	0.02		
Hematocrit** (**%)	− 0.41 (− 0.79 to 0.03)	0.04	− 0.05 (− 0.35 to 0.18)	0.50
eGFR (mL/min/1.73m^2^)	− 0.06 (− 0.16 to − 0.03)	0.33		
eGFR slope (mL/min/1.73m^2^/year)	− 1.14 (− 1.33 to − 0.96)	< 0.001	− 0.67 (− 1.18 to − 0.75)	< 0.001
Urinary protein (g/gCr)	− 0.14 (− 0.89 to 1.17)	0.78		
U-RBC (cells/HPF)	0.02 (− 0.10 to 0.15)	0.69		
Prescription rate of RAS inhibitors (%)	1.73 (− 2.27 to 5.74)	0.39		
Prescription rate of ULD (%)	− 1.32 (− 5.02 to 2.38)	0.48	− 0.09 (− 4.23 to 1.01)	0.23
Change in BMI (kg/m^2^)	− 2.18 (− 3.64 to − 0.71)	0.004	0.00 (− 1.03 to 1.05)	0.99
Change in HbA1c (%)	1.49 (− 0.54 to 3.52)	0.15		
Change in SBP (mmHg)	0.00 (− 0.12 to 0.13)	0.98		
Change in LDL-C (mg/dL)	− 0.07 (− 0.15 to 0.02)	0.11		
Change in uric acid (mg/dL)	− 4.42 (− 5.89 to − 2.95)	< 0.001	− 0.26 (−3.34 to − 0.92)	< 0.001
Change in hematocrit (%)	− 0.17 (− 0.62 to 0.27)	0.44		
Change in urinary protein (%)	0.01 (− 0.02 to 0.03)	0.56	0.04 (− 0.01 to 0.02)	0.58
Change in U-RBC (cell/HPF)	− 0.08 (− 0.22 to 0.07)	0.30		

*β*, unstandardized regression coefficient; standardized *β*, regression coefficient after standardization of all variables, allowing comparison of effect sizes; 95%CI, 95% confidence intervals

*SGLT2* inhibitor sodium–glucose cotransporter 2 inhibitor, *DM* diabetes mellitus, *BMI* body mass index, *HbA1c* hemoglobin A1c, *SBP* systolic blood pressure, *LDL-C* low-density lipoprotein cholesterol, *eGFR* estimated glomerular filtration rate, *U-RBC* urinary red blood cells per high-power field, *RAS* renin–angiotensin system, *ULD* uric acid-lowering drug

In the multivariable regression analysis, pre-treatment eGFR slope (standardized *β* =  − 0.67; 95% CI − 1.18 to − 0.75; *p* < 0.001) and change in serum uric acid (standardized *β* = −0.26; 95% CI − 3.34 to − 0.92; *p* < 0.001) remained independently associated with ΔeGFR slope after adjustment for age, sex, baseline hematocrit, use of urate-lowering drugs, change in BMI, and change in urinary protein (Table 3). Specifically, a steeper pre-treatment eGFR slope and a greater reduction in serum uric acid were significantly associated with greater improvement in ΔeGFR slope. None of the other covariates included in the model were significantly associated with ΔeGFR slope. In addition, multivariable regression analysis demonstrated that the change in serum uric acid remained significantly associated with ΔeGFR slope even after adjustment for baseline eGFR slope and other covariates (Table 3), suggesting that the observed association cannot be explained solely by statistical regression toward the mean.

**Fig. 3:**
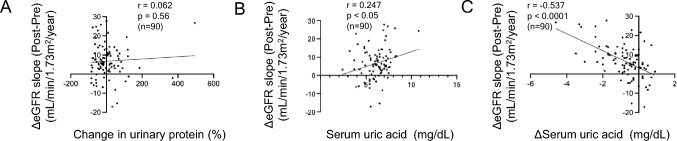
Association between improvement in eGFR slope before and after SGLT2 inhibitor therapy and changes in urinary protein and serum uric acid. **A** Correlation between the change in eGFR slope and the change in urinary protein. **B** Correlation between the change in eGFR slope and baseline serum uric acid levels. **C** Correlation between the change in eGFR slope and the change in serum uric acid levels

Consistent with the multivariable regression analysis, linear regression analyses demonstrated no significant association between ΔeGFR slope and the percentage change in urinary protein (*r* = 0.062, *p* = 0.56; Fig. 3A. Baseline serum uric acid showed a weak but statistically significant correlation with ΔeGFR slope (*r* = 0.247, *p* < 0.05; Fig. 3B. In contrast, the change in serum uric acid demonstrated a significant and moderate inverse correlation with ΔeGFR slope (*r* =  − 0.537, *p* < 0.0001; Fig. 3C. These findings suggest that improvement in ΔeGFR slope was more strongly associated with changes in serum uric acid than with changes in urinary protein in this cohort.

## Discussion

In this single-center real-world CKD cohort, initiation of an SGLT2 inhibitor was associated with a significant improvement in post-treatment eGFR slope compared with the pre-treatment period, indicating a marked attenuation of kidney function decline. This renoprotective effect was consistently observed across CKD risk categories and was not statistically associated with baseline urinary protein levels or with treatment-related changes in urinary protein in this cohort. By evaluating within-patient changes in eGFR trajectory (ΔeGFR slope), our study complements evidence from large randomized-controlled trials (RCTs) and meta-analyses, suggesting that SGLT2 inhibitors provide renal benefits that are not solely explained by reductions in albuminuria [3, 12, 13]. These findings support the clinical value of SGLT2 inhibitors for kidney protection in routine practice, including among patients with advanced CKD or those with modest urinary protein responses. In addition, reductions in serum uric acid were independently associated with improvement in eGFR slope, suggesting that uric acid lowering may represent a clinical correlate of the renoprotective effects of SGLT2 inhibition.

Because ΔeGFR slope is mathematically influenced by baseline slope values, regression to the mean must be considered when interpreting within-patient comparisons. However, improvement in post-treatment eGFR slope was observed in both subgroups defined by baseline slope relative to the cohort mean, indicating that the observed effect was not confined to patients with steep baseline decline. Moreover, the association between uric acid reduction and ΔeGFR slope remained significant after adjustment for baseline slope in multivariable analysis. These findings suggest that the observed improvement is unlikely to be explained solely by statistical tendency, although residual regression-to-the-mean effects cannot be completely excluded. Importantly, while major RCTs, such as DAPA-CKD and EMPA-KIDNEY, demonstrated attenuation of eGFR decline compared with placebo, these trials did not evaluate within-patient pre- and post-treatment changes in eGFR slope. A recent analysis from the Japanese J-CKD-DB study reported significant improvement in eGFR slope using a similar pre–post-design, although that study was limited to patients with diabetes [14]. Our findings, therefore, provide complementary real-world evidence supporting the renoprotective effects of SGLT2 inhibitors in both diabetic and non-diabetic CKD populations.

Another important observation in this study concerns the relationship between within-patient changes in proteinuria and the ΔeGFR slope. Reduction in proteinuria has traditionally been regarded as a key therapeutic target in CKD management, because persistent proteinuria contributes to tubular injury, nephron loss, and progressive kidney function decline [10]. However, randomized-controlled trials of SGLT2 inhibitors have indicated that the attenuation of eGFR decline with these agents cannot be fully explained by reductions in proteinuria alone [12, 13]. In the present study, we evaluated changes in eGFR slopes before and after treatment within the same individuals, allowing assessment of treatment-related changes in kidney function trajectory in a real-world clinical setting. In this cohort, improvement in the ΔeGFR slope was not significantly associated with the degree of change in proteinuria. Although the observational design and limited sample size preclude definitive conclusions, these findings are consistent with prior reports, suggesting that SGLT2 inhibitors may confer renoprotective effects through mechanisms that extend beyond proteinuria reduction. Our results are also consistent with a recent single-center cohort study of patients with IgA nephropathy, in which treatment with SGLT2 inhibitors was associated with improvement in the rate of eGFR decline despite no significant reduction in proteinuria [15]. Taken together, these observations support the possibility that SGLT2 inhibitors may improve eGFR trajectories even when changes in proteinuria are modest.

Our analysis also suggests that urinary protein changes alone may be insufficient as a surrogate marker for predicting the renal benefits of SGLT2 inhibitors. Previous studies have explored alternative clinical indicators of treatment response. For example, a post hoc analysis of the EMPA-REG OUTCOME trial identified reductions in HbA1c and blood pressure and increases in hematocrit and serum free fatty acids as predictors of improved renal outcomes [16]. In the present study, however, these variables were not independently associated with improvement in eGFR slope. Although reductions in serum uric acid were independently associated with slope improvement, this relationship should be interpreted as hypothesis-generating rather than causal. Uric acid reduction may reflect broader metabolic or hemodynamic effects of SGLT2 inhibition rather than acting as a direct mediator of renoprotection.

Several limitations should be considered when interpreting our findings. First, this retrospective single-center study included a relatively small sample size, which may limit generalizability and leave residual confounding. Second, hard kidney endpoints such as kidney replacement therapy were not systematically evaluated, and the post-treatment observation period was limited to approximately 12 months. Third, the absence of a statistically significant reduction in urinary protein should not be interpreted as evidence that SGLT2 inhibitors lack antiproteinuric effects. The median baseline urinary protein level in our cohort was lower than that reported in large RCTs such as DAPA-CKD [2], which may have limited the power to detect treatment-related reductions in urinary protein. Despite these limitations, the within-patient slope-based analysis provided a sensitive approach for evaluating treatment-related changes in the trajectory of kidney function. Taken together, these findings suggest that monitoring changes in eGFR slope may provide a practical approach for evaluating the renal response to SGLT2 inhibitor therapy in real-world clinical settings.

## Conclusion

In this real-world CKD cohort, SGLT2 inhibition significantly attenuated the decline in eGFR. This improvement in eGFR slope was observed across patients with varying levels of baseline urinary protein and was not statistically associated with treatment-related reductions in urinary protein. Furthermore, within-patient ΔeGFR slope analysis provided a sensitive measure for detecting therapeutic benefits and may serve as a practical tool for monitoring treatment response in routine clinical care. Collectively, our findings support the use of SGLT2 inhibitors for renal protection across a broad range of CKD patients and highlight the clinical importance of developing prognostic markers for renal outcomes that are not solely dependent on changes in urinary protein.
